# Familial Hypomagnesemia With Hypercalciuria and Nephrocalcinosis: A Case Report of Compound Heterozygous Variants in the *CLDN16* Gene Inherited From Symptomatic Heterozygous Carriers

**DOI:** 10.1159/cnd/abxag006

**Published:** 2026-10-07

**Authors:** Monica Ceol, Elena Sgrò, Franca Anglani, Giovanna Priante, Lucia Federica Stefanelli, Martina Cacciapuoti, Leonardo Salviati, Federico Nalesso, Dorella Del Prete

**Affiliations:** a Nephrology Unit, Department of Medicine-DIMED, University of Padua, Padua, Italy; b Clinical Genetics Unit, Department of Women and Children’s Health, University of Padua, Padua, Italy

**Keywords:** Genetic disease, Case report, Familial hypomagnesemia with hypercalciuria and nephrocalcinosis, *CLDN16*, Next-generation sequencing

## Abstract

**Introduction:**

Familial hypomagnesemia with hypercalciuria and nephrocalcinosis (FHHNC) is a rare autosomal recessive disorder characterized by renal magnesium wasting, hypercalciuria, and early onset of nephrocalcinosis, complicated by progressive chronic renal failure in childhood or adolescence. The disorder is caused by *CLDN16* or *CLDN19* genes’ pathogenic variants.

**Case Presentation:**

We describe a 22-year-old female patient with a clinical diagnosis of FHHNC in infancy. Detailed clinical evaluation revealed a complex phenotype with early nephrocalcinosis, recurrent self-resolving renal colic episodes, persistent hypomagnesemia, and secondary hyperparathyroidism (HPP). High oral doses of magnesium were required to treat the FHHNC, causing significant gastrointestinal side effects, particularly diarrhea, leading to further magnesium losses. Upon transfer to our adult nephrology unit, the proband’s blood was subjected to genetic analysis using next-generation sequencing with multigene panel testing.

**Results:**

Genetic analysis of the proband revealed compound heterozygous variants in the *CLDN16* gene: a novel splice-site variant NC_000003.12 (NM_006580.4):c.382+1G>C in the canonical donor splice consensus sequence, classified as likely pathogenic inherited from the mother with HPP phenotype; a rare missense variant NM_006580.4:c.500G>C p.(Trp167Ser) inherited from the father with hypercalciuria. Due to poor tolerability of conventional oral magnesium salts, she was switched to a Sucrosomial magnesium formulation, which significantly improved her gastrointestinal tolerance and adherence.

**Conclusion:**

The novel splice-site variant c.382+1G>C and the previously unreported missense variant p.(Trp167Ser) in *CLDN16* expand the mutational spectrum associated with FHHNC. Regarding the mother’s phenotype, a symptomatic carrier of a recessive autosomal disease, our finding suggests the importance of genotype-phenotype analysis in identifying mild forms of FHHNC.

## Introduction

Familial hypomagnesemia with hypercalciuria and nephrocalcinosis (FHHNC, MIM #248250) is a rare autosomal recessive disorder characterized by renal magnesium wasting, hypercalciuria, and early onset of nephrocalcinosis, complicated by progressive chronic renal failure in childhood or adolescence. It is caused by pathogenic variants in the *CLDN16* (MIM 603959) gene, and less frequently in the *CLDN19* (MIM 610036) gene, that encode, respectively, claudins 16 and 19, which play a crucial role in the paracellular reabsorption of magnesium and calcium in the thick ascending limb of Henle’s loop [1].

FHHNC can be detected perinatally, but the median age at diagnosis is 5 years [1]. The disease is present with clinical symptoms such as polyuria, polydipsia, and, less frequently, urinary tract infections and lithiasis. Chronic hypomagnesemia contributes to hyperparathyroidism (HPP), further exacerbating calcium excretion and promoting nephrocalcinosis and renal dysfunction [2]. Ultrasonography reveals characteristic calcifications in the neonatal kidneys, sometimes described as having a flower petal-like appearance.

While several pathogenic variants in *CLDN16* and *CLDN19* have been reported, novel variants continue to emerge [3]. Supportive therapy with magnesium and thiazide diuretics is currently used to delay the progression of chronic kidney disease to the end stage [4].

We describe the case of a female patient with FHHNC harboring two previously unreported *CLDN16* variants*,* whose family history and clinical phenotype may add new insights into this rare disease.

## Case Report

### Clinical History

A 22-year-old female was diagnosed with FHHNC aged 14 months on the basis of biochemical analyses performed during a hospitalization for a urinary tract infection, which revealed severe hypomagnesemia and hypercalciuria. Renal ultrasound showed extensive nephrocalcinosis. Genetic testing was unavailable at the time. In early childhood, she experienced a seizure preceded by paresthesia, with serum magnesium levels around 0.20 mmol/L.

Between the ages of 15 and 20 years, she had multiple episodes of recurrent renal colic and nephrolithiasis. Laboratory follow-up showed persistent hypomagnesemia, hypercalciuria, and elevated parathyroid hormone (PTH) levels, indicative of secondary HPP. Serial ultrasounds revealed cortical thinning and medullary hyperechogenicity. A resistivity index of approximately 0.68 bilaterally was reported, with greater involvement of the right kidney, correlating with the predominance of right-sided renal colic episodes. Ultrasound follow-up since 2021 has shown a slight deterioration in the appearance of the kidneys, with marked medullary hyperechogenicity and bilateral cortical thinning. The kidneys measured 10 cm in length and showed multiple bilateral pyramidal/medullary cysts (maximum diameter 2 cm) (Fig. 1).

**Fig. 1.**
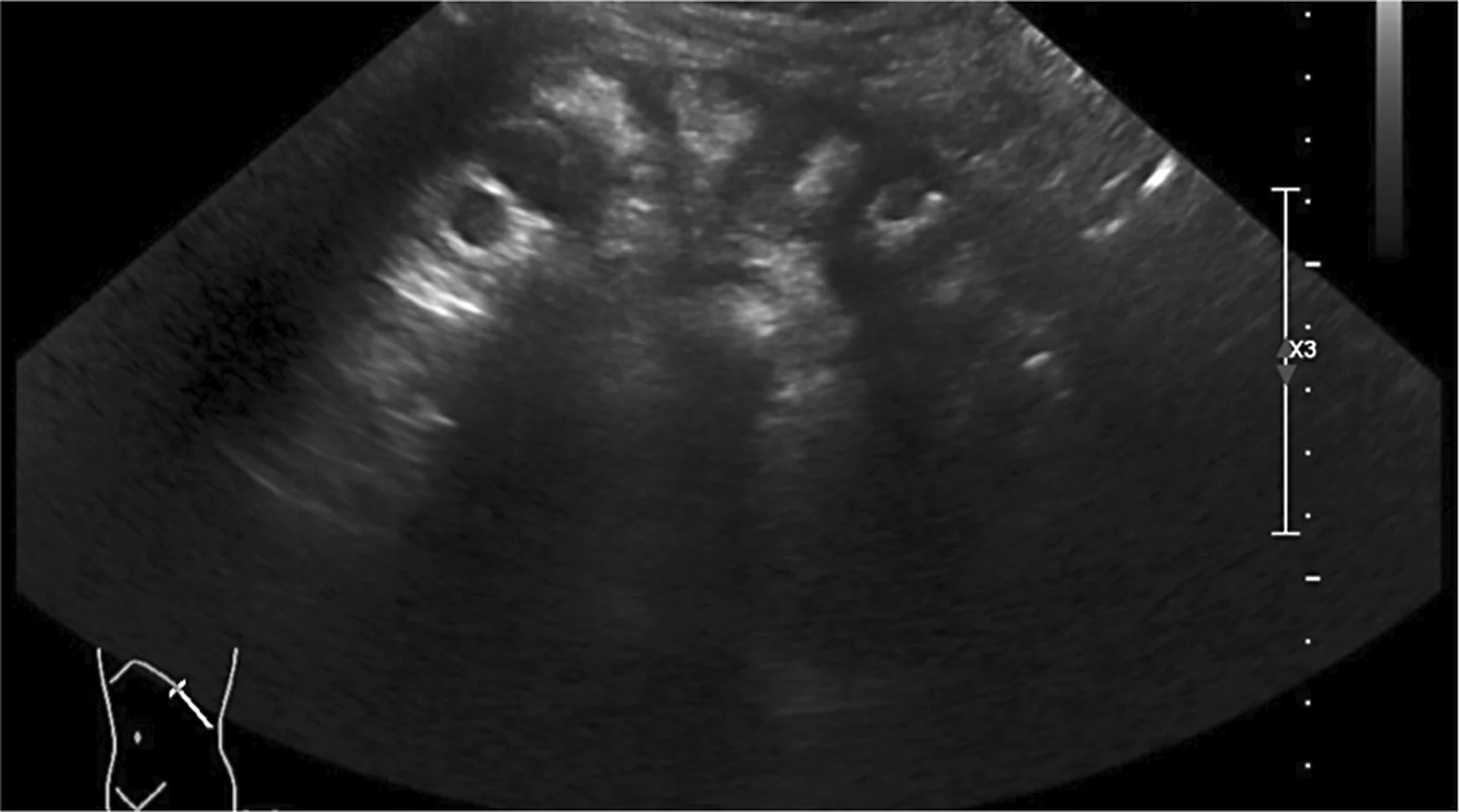
Representative image of the proband’s ultrasound: kidney with marked medullary hyperechogenicity and cortical thinning. Within the pyramids, multiple cystic images with transonic content of centimetric dimensions.

Since childhood, she has been prescribed magnesium supplements in various formulations. However, adherence has been poor due to severe gastrointestinal side effects (chronic diarrhea, abdominal discomfort) leading to malabsorption. She was stabilized with a daily dose of 8 g of magnesium glycosulfate-based galenic preparations (eight sachets per day), resulting in asymptomatic hypomagnesemia [5]. To mitigate nephrocalcinosis, she was also prescribed a thiazide diuretic combined with amiloride 5/50 mg, half a tablet twice daily. Despite amiloride treatment, she needed daily potassium supplementation with 600 mg potassium chloride. We have no information regarding citrate supplementation. The patient’s renal function remained stable, with an estimated glomerular filtration rate >60 mL/min, with persistent microalbuminuria. Her blood pressure was consistently low (90/60 mmHg), and she experienced episodic paresthesia, tension-type headaches, and vitamin D deficiency. A DEXA scan indicated normal bone mineral density. Upon transfer to our adult nephrology unit, genetic analyses of blood sample from the proband and her parents were performed.

Due to poor tolerability of conventional oral magnesium salts, the patient was switched to a Sucrosomial magnesium formulation, which significantly improved her gastrointestinal tolerance and adherence. Within 6 months, serum magnesium levels had stabilized, PTH levels had declined, and the potassium supplementation was discontinued. The patient reported an overall improvement, reduced gastrointestinal symptoms, and no electrolyte imbalance-related events. Her BMI increased from 17.7 to 19 kg/m^2^. Laboratory assessments confirmed stable renal function and stable serum magnesium levels around 0.48 mmol/L. A parathyroid ultrasound in 2024 showed no signs of primary HPP. She is currently continuing treatment with three sachets per day of Sucrosomial magnesium dissolved in 500 mL of water, taken throughout the day, along with vitamin D supplementation to manage secondary HPP.

The thiazide diuretic plus amiloride was discontinued during the summer due to two episodes of symptomatic hypotension (60/45 mmHg). Urinary calcium levels consequently rose slightly from a median of 180 mg/24 h to 220 mg/24 h, but there were no further episodes of renal colic. In addition, magnesium wasting was reduced after discontinuation of the thiazide diuretic, from a median of 296.46 mg/24 h to a median of 196.83 mg/24 h. Table 1 summarizes the main clinical parameters during follow-up. Vitamin D needed careful dose adjustment: the initial 10,000 IU/week triggered recurrent nephrolithiasis, so it was lowered to 8,750 IU/week, thereby stabilizing vitamin D levels and preventing further colic episodes for over 12 months. Regular physical activity was encouraged to support bone health.

**Table 1.** Proband’s Biochemical Data During Follow-Up Before and After Sucrosomial Magnesium Treatment

Normal values	Before	After
Mg mmol/L (0.77–1.03)	0.61 (0.53–0.67)	0.48 (0.35–0.54)
Ca mmol/L (2.12–2.62)	2.5 (2.47–2.54)	2.40 (2.01–2.42)
P mmol/L (0.8–1.45)	0.96 (0.77–1.29)	1.05 (0.98–1.11)
PTH pg/mL (6.5–36.8)	222.5 (189–239.3)	165 (158–172)
Vit D nmol/L (19.9–199.6)	47.75 (30.25–65.25)	55.75 (49.75–61.75)
uCa mg/24 h (30–250)	180 (174–226.2)	220 (218–232)
uMg mg/24 h (72.9–121.5)	296.46 (232.06–311.04)	196.83 (189.05–282.48)
Creatinine µmol/L (44–90)	84.5 (83.6–90.6)	88 (78.3–91)
eGFR mL/min	>60	>60
uProt g/24 h (<0.2)	0.32 (0.28–0.36)	0.36 (0.26–0.39)

Values are shown as median (min–max).

eGFR, estimated glomerular filtration rate.

### Genetic Analysis

Next-generation sequencing was performed on the Illumina NextSeq 2000 platform using a custom Agilent-SureSelect panel of genes associated with renal tubulopathies, including *CLDN16*, *CLDN19*, *CNNM2*, *EGF*, *FXYD2*, *KCNA1*, and *TRPM6*. Data were processed with Illumina DRAGEN 4.2.4 and analyzed further with Emedgene 35.0.3, with reads aligned to the human reference genome (GRCh38/hg38). Variants were classified according to the interpretation guidelines of the American College of Medical Genetics and Genomics (ACMG) [6]. Two heterozygous variants in the *CLDN16* gene were identified. A novel splice-site variant NC_000003.12 (NM_006580.4):c.382+1G>C (Minor Allele Frequency MAF = 0) in the canonical donor splice consensus sequence was classified as likely pathogenic and not previously described, while the rare missense variant NM_006580.4:c.500G>C p.(Trp167Ser) (rs1453219704) (MAF <0.01% GnomAD v4.1) was classified as a variant of uncertain significance. The missense variant substitutes a highly conserved aromatic residue with a polar amino acid. Pathogenicity prediction tools predicted a deleterious effect on protein structure and function, impairing ion selectivity and protein stability, and identified the variant as potentially pathogenic. This variant has not been previously reported in the literature. The segregation study revealed the heterozygous variant c.382+1G>C on the maternal allele, and the heterozygous p.(Trp167Ser) variant on the paternal allele (Fig. 2)

**Fig. 2.**
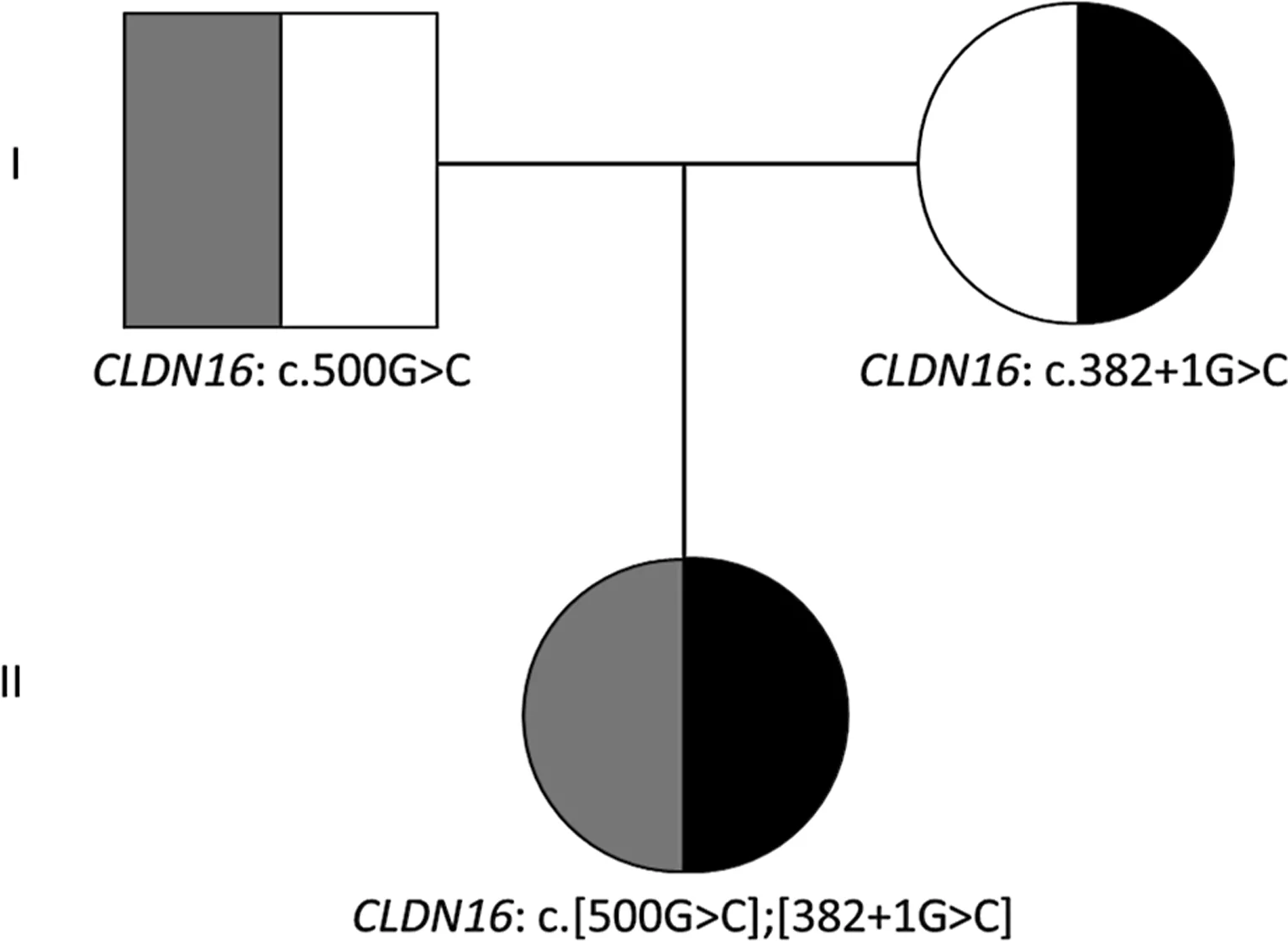
Family pedigree. Compound heterozygosity was found in the patient for the *CLDN16* variants c.[500G>C];[382+1G>C] inherited from her parents.

### Family History

The proband’s mother developed renal calculi at the age of 45 following a hysterectomy with adnexectomy. She experienced significant stone burden with unilateral (left-sided) renal colic, and scintigraphy demonstrated only 18% residual renal function in the affected kidney. Multiple renal stenting procedures were performed, although these were later removed due to stone formation over the stents. Elevated PTH levels were observed in the mother. Parathyroid ultrasonography, followed by choline PET imaging, identified two hyperfunctioning nodules in the posterior portion of the thyroid. She has two younger brothers who are in good health, but her father had renal calculi, although this was never thoroughly investigated. The proband’s father had an episode of renal calculi at 60 years of age, with spontaneous passage of a struvite stone in the context of a urinary tract infection secondary to prostatic hypertrophy. Subsequent evaluations revealed hypercalciuria (331.2 mg/24 h). Table 2 shows the genotype-phenotype analysis of the family members. The CARE Checklist has been completed by the authors for this case report, attached as online supplementary material (for all online suppl. material, see https://doi.org/10.1159/cnd/abxag006).

**Table 2.** Genotype-Phenotype Analysis of the Family Members

Proband, *CLDN16*: c.[382+1G>C]; [500G>C]	Mother’s proband, *CLDN16*: c.382+1G>C	Father’s proband, *CLDN16*: c.500G>C
Hypercalciuria	Hypercalciuria	Hypercalciuria
Nephrolithiasis	Nephrolithiasis	​
Nephrocalcinosis	Secondary hyperparathyroidism	​
Hypomagnesemia	CKD	​
Secondary hyperparathyroidism	​	​

CKD, chronic kidney disease.

## Discussion

Genetic analysis of the proband revealed compound heterozygous variants in the *CLDN16* gene. The patient’s clinical history aligns with the typical FHHNC phenotype. Early-onset nephrocalcinosis, persistent hypomagnesemia resistant to conventional oral magnesium formulations, recurrent renal colic with spontaneous passage of calculi, and secondary endocrine abnormalities (elevated PTH, vitamin D deficiency, and hypophosphatemia) are consistent with functional impairment of claudin-16. Canonical splice-site variants are often presumed to cause loss-of-function and are assigned very strong evidence of pathogenicity. A deleterious effect on splicing was predicted for the splice-site variant c.382+1G>C, potentially resulting in a nonfunctional protein product. Interpretation of the presence in trans of the missense variant p.(Trp167Ser), classified as variant of uncertain significance according to ACMG classification, poses some challenges. Although it is located within a domain critical for maintaining tight junction integrity where other similar missense variants have been reported in clinical databases, no previous cases involving this specific variant have been described yet. Although segregation data and in silico predictions support the possibility that p.(Trp167Ser) variant contributes to the phenotype, these findings do not establish pathogenicity and, in the absence of functional evidence, its role in disease remains uncertain.

Several case reports have described patients with only one variant of biallelic Mendelian diseases displaying mild phenotype [7–9]. FHHNC carriers are usually completely asymptomatic, but some studies have reported subtle or partial phenotypic expressions among heterozygous carriers. The clinical manifestations observed in the proband’s parents are noteworthy: the mother, heterozygous for the *CLDN16* splice-site variant, presented with hypercalciuria, nephrolithiasis, and HPP, while the father, heterozygous for the rare, potentially deleterious, missense variant, presented only with hypercalciuria. Although the multifactorial nature of nephrolithiasis might be a confounding factor, we speculate that the parent’s phenotype might be associated with *CLDN16* variants, that is, with their carrier status. This interpretation is consistent with the findings of Weber et al. [10], who suggested that individuals who are heterozygous for pathogenic variants of *CLDN16* may have an increased risk of developing nephrolithiasis. They showed that in about 50% of families in which FHHNC segregates with *CLDN16* mutations, unaffected heterozygous family members presented with nephrolithiasis and/or hypercalciuria. Naeem et al. [11] reported that nephrolithiasis was present in 50% of heterozygous carriers of *CLDN19* mutations. The association of single nucleotide variants in claudin genes, such as *CLDN14* and *CLDN2*, with renal lithiasis has been recently demonstrated by genome-wide association studies (GWAS) [12–14] further corroborating our findings.

The phenotype observed in the mother, who carries the novel splicing variant c.382+1G>C, is of interest. The presence of HPP associated with hypercalciuria and nephrolithiasis leads us to hypothesize that the novel splice-site variant in the *CLDN16* gene might explain her phenotype, supporting the possibility of mild phenotypic expression of FHHNC in some heterozygous carriers [9]. However, due to the presence of elevated PTH combined with positive imaging findings, we cannot determine with any certainty whether HPP is secondary to the *CLDN16* gene variant.

High oral doses of magnesium are required to treat FHHNC, often with significant gastrointestinal side effects, particularly diarrhea, leading to further magnesium losses. A recently developed formulation, Sucrosomial magnesium, enhances absorption and reduces side effects. The different magnesium formulations used in treating FHHNC are listed in Table 3. Unlike conventional magnesium salts, Sucrosomial magnesium encapsulates the mineral in a phospholipid matrix, facilitating its transport across the intestinal epithelium via endocytosis rather than relying on passive diffusion. This mechanism increases bioavailability and avoids the limitations of traditional magnesium supplements, which often cause osmotic diarrhea due to unabsorbed magnesium in the gut lumen [2]. With its innovative formulation, Sucrosomial magnesium has been shown to be effective both in the rare genetic tubulopathies Bartter’s and Gitelman’s syndromes, and in kidney transplant patients [15]. There is currently no specific therapy for FHHNC. Typical management includes magnesium supplementation, high fluid intake, thiazide and alkali citrate therapy, and potassium supplementation if needed. One crucial aspect often overlooked in the literature is the impact of chronic hypomagnesemia and gastrointestinal intolerance on nutritional status and body weight. Persistent diarrhea can be both physically debilitating and socially distressing, causing embarrassment and limiting daily activities. In our patient, the switch to Sucrosomial magnesium was associated with a significant weight gain (from a BMI of 17.7 to 19 kg/m^2^), along with improved well-being and nutrient absorption, although serum magnesium levels did not increase significantly.

**Table 3.** Comparison of Magnesium Formulations Used in Italy

Formulation	Approx. elemental Mg content	Bioavailability	Notable side effects/notes
Magnesium oxide	∼241 mg of Mg/standard tablet	Extremely low	Can cause significant gastrointestinal distress (diarrhea). Often poorly absorbed
Magnesium citrate	Highly variable (e.g., ∼90–300 mg per dose)	Good/moderate	Mild laxative effect. Better solubility in water than many inorganic salts; sometimes used to help reduce risk of nephrolithiasis
Magnesium sulfate	∼240 mg per 10 mL	Moderate to high	Can cause gastrointestinal distress, especially in high doses. Used intravenously in acute settings
Magnesium pidolate	∼370 mg of Mg daily with 3 vials	Moderate (not extensively quantified)	Frequently reported GI side effects (e.g., diarrhea) and intolerance
Magnesium glycosulfate	Varies by preparation; for example, up to several grams/day	Not specifically quantified	Pronounced GI effects (diarrhea, abdominal cramps) and malabsorption
Magnesium bisglycinate	Varies; often ∼100–200 mg elemental Mg per dose	Considered high	Amino acid-chelated form. Generally well tolerated, with fewer laxative effects compared to inorganic salts. Recommended when GI side effects limit higher doses
Sucrosomial magnesium	∼375 mg of Mg per sachet	High	Sucrosomial technology encapsulates magnesium oxide in a phospholipid membrane, enhancing absorption and reducing gastro intestinal discomfort

Our observation, however, regards a single case of FHHNC, and further scientific evidence specific to this condition is needed.

## Conclusions

Identification of the novel canonical splice-site variant NC_000003.12 (NM_006580.4):c.382+1G>C and the rare missense variant p.(Trp167Ser) in *CLDN16* correlates with the severe electrolyte and renal phenotype observed in the patient, and expands the mutational spectrum associated with FHHNC.

Of particular interest is the phenotype of the proband’s mother, who is a symptomatic carrier of a recessive autosomal disease. Although our findings are based on observations of a single family, they suggest caution in determining whether an HPP is primary or secondary, and highlight the importance of genetic analysis.

Sucrosomial magnesium appeared to be better tolerated and was associated with improved adherence in this patient. Its greater bioavailability and reduced gastrointestinal side effects improve treatment adherence and overall patient outcomes.

## Statements of Ethics

This study was performed in accordance with the Declaration of Helsinki. Ethical approval was not required for this study as per Article 5 of the Code of Conduct approved by the University Hospital of Padua. Written informed consent to publish the details of their medical case was obtained from the patients.

## Conflict of Interest Statement

The authors have no conflict of interest to declare.

## Funding Sources

This study was not supported by any sponsor or funding body.

## Author Contributions

Dorella Del Prete and Monica Ceol wrote the manuscript. Franca Anglani, Federico Nalesso, and Giovanna Priante revised the manuscript. Leonardo Salviati and Monica Ceol acquired and analyzed the genetic data. Franca Anglani discussed the genetic data. Dorella Del Prete, Elena Sgrò, Martina Cacciapuoti, and Lucia Federica Stefanelli supervised the clinical case and clinical data. All authors read and approved the final manuscript.

## Data Availability

The data supporting the findings of this study are not publicly available for reasons of privacy, but are available from the corresponding author upon request.

## References

[1] Vall-Palomar M , Madariaga L, Ariceta G. Familial hypomagnesemia with hypercalciuria and nephrocalcinosis. Pediatr Nephrol. 2021;36(10):3045–55. 10.1007/s00467-021-04968-2 33595712

[2] Adomako EA , Yu ASL. Magnesium disorders: core curriculum 2024. Am J Kidney Dis. 2024;83(6):803–15. 10.1053/j.ajkd.2023.10.017 38372687

[3] Rungrasameviriya P , Santilinon A, Atichartsintop P, Hadpech S, Thongboonkerd V. Tight junction and kidney stone disease. Tissue Barriers. 2024;12(1):2210051. 10.1080/21688370.2023.2210051 PMC10832927 37162265

[4] Eltan M , Yavas Abali Z, Turkyilmaz A, Gokce I, Abali S, Alavanda C, . Familial hypomagnesemia with hypercalciuria and nephrocalcinosis due to CLDN16 gene mutations: novel findings in two cases with diverse clinical features. Calcif Tissue Int. 2022;110(4):441–50. 10.1007/s00223-021-00928-y 34761296

[5] Bennati G , Cirino M, Benericetti G, Maximova N, Zanier M, Pigato F, . Compounded effervescent magnesium for familial hypomagnesemia: a case report. Pharmaceuticals. 2023;16(6):785. 10.3390/ph16060785 PMC10305453 37375733

[6] Richards S , Aziz N, Bale S, Bick D, Das S, Gastier-Foster J, . Standards and guidelines for the interpretation of sequence variants: a joint consensus recommendation of the American College of Medical Genetics and Genomics and the Association for Molecular Pathology. Genet Med. 2015;17(5):405–24. 10.1038/gim.2015.30 PMC4544753 25741868

[7] Barton AR , Hujoel MLA, Mukamel RE, Sherman MA, Loh P-R. A spectrum of recessiveness among Mendelian disease variants in UK Biobank. Am J Hum Genet. 2022;109(7):1298–307. 10.1016/j.ajhg.2022.05.008 PMC9300759 35649421

[8] Heyne HO , Karjalainen J, Karczewski KJ, Lemmelä SM, Zhou W; FinnGen; . Mono- and biallelic variant effects on disease at biobank scale. Nature. 2023;613(7944):519–25. 10.1038/s41586-022-05420-7 PMC9849130 36653560

[9] Kalyta K , Stelmaszczyk W, Szczęśniak D, Kotuła L, Dobosz P, Mroczek M. The spectrum of the heterozygous effect in biallelic Mendelian diseases-the symptomatic heterozygote issue. Genes. 2023;14(8):1562. 10.3390/genes14081562 PMC10454578 37628614

[10] Weber S , Schneider L, Peters M, Misselwitz J, Rönnefarth G, Böswald M, . Novel paracellin-1 mutations in 25 families with familial hypomagnesemia with hypercalciuria and nephrocalcinosis. J Am Soc Nephrol. 2001;12(9):1872–81. 10.1681/ASN.V1291872 11518780

[11] Naeem M , Hussain S, Akhtar N. Mutation in the tight-junction gene claudin 19 (CLDN19) and familial hypomagnesemia, hypercalciuria, nephrocalcinosis (FHHNC) and severe ocular disease. Am J Nephrol. 2011;34(3):241–8. 10.1159/000330854 21791920

[12] Negri AL , Del Valle EE. Role of claudins in idiopathic hypercalciuria and renal lithiasis. Int Urol Nephrol. 2022;54(9):2197–204. 10.1007/s11255-022-03119-2 35084652

[13] Curry JN , Saurette M, Askari M, Pei L, Filla MB, Beggs MR, . Claudin-2 deficiency associates with hypercalciuria in mice and human kidney stone disease. J Clin Investig. 2020;130(4):1948–60. 10.1172/JCI127750 PMC7108907 32149733

[14] Arcidiacono T , Simonini M, Lanzani C, Citterio L, Salvi E, Barlassina C, . Claudin-14 gene polymorphisms and urine calcium excretion. Clin J Am Soc Nephrol. 2018;13(10):1542–9. 10.2215/CJN.01770218 PMC6218816 30232134

[15] Stefanelli LF , Alessi M, Bertoldi G, Rossato V, Di Vico V, Nalesso F, . Calcineurin-inhibitor-induced hypomagnesemia in kidney transplant patients: a monocentric comparative study between sucrosomial magnesium and magnesium pidolate supplementation. J Clin Med. 2023;12(3):752. 10.3390/jcm12030752 PMC9917957 36769401

